# Potential for Detection of Safety Signals for Over-the-Counter Medicines Using National ADR Spontaneous Reporting Data: The Example of OTC NSAID-Associated Gastrointestinal Bleeding

**DOI:** 10.3390/pharmacy8030174

**Published:** 2020-09-17

**Authors:** Elina Amatya, Romano Fois, Kylie A. Williams, Lisa G. Pont

**Affiliations:** 1 eHealth, Chatswood, NSW 2067, Australia; sojelina@gmail.com; 2 Ferring Pharmaceuticals, Pymble, NSW 2073, Australia; romanof015@gmail.com; 3 Discipline of Pharmacy, Graduate School of Health, University of Technology Sydney (UTS), Sydney, NSW 2007, Australia; kylie.williams@uts.edu.au

**Keywords:** pharmacovigilance, OTC medicines, signal detection, NSAIDs, gastrointestinal bleeding

## Abstract

One post-marketing surveillance challenge for many regulatory authorities is access to information regarding the safety of over-the-counter (OTC) medicines. National spontaneous adverse drug reaction (ADR) report data represent a rich potential data source for the detection of safety signals associated with OTC medicines, yet little is known regarding the possibility of detecting safety signals for OTC medicines within these datasets. The aim of this study was to evaluate the potential for detecting safety signals for OTC medicines in National ADR spontaneous reporting data, using OTC non-steroidal anti-inflammatory drugs (NSAIDs) and gastrointestinal bleeding as an example. Data from the Australian Adverse Drug Reactions System (ADRS) dataset (1971–2008) and the Canadian Vigilance Adverse Reaction Online Database (VAROD) (1965–2013) were used to explore the feasibility of using spontaneous reporting data, exploring the association between gastrointestinal bleeding and the use of OTC NSAIDs. Safety signals were examined using disproportionality analyses and reporting odds ratios calculated. After adjusting for age, gender, medications known to increase the risk of bleeding, and medications used for the management of conditions associated with an increased risk of bleeding, a two-fold increase in the risk of gastrointestinal (GI) bleeding with OTC NSAID was observed within each dataset. This study demonstrates that spontaneous ADR reporting data can be used in pharmacovigilance to monitor the safety of OTC medicines.

## 1. Introduction

Internationally, data from spontaneous reports of suspected adverse drug reactions (ADRs) play a critical role in pharmacovigilance and ensuring the safety of medicines. The World Health Organization defines adverse drug reactions as “a response to a drug which is noxious and unintended, and which occurs at doses normally used in man for the prophylaxis, diagnosis, or therapy of disease, or for the modification of a physiological function” [1], and the detection of new ADRs via the detection of safety signals is an important aspect of pharmacovigilance. While there are a number of methods used internationally to monitor adverse drug reactions, including prescription event monitoring and active surveillance, spontaneous reporting of ADRs is considered the best practice for safety signal detection [2] and it is the most widely used pharmacovigilance method for monitoring the safety of marketed medicines internationally [3].

One challenge for many regulatory authorities in the post-marketing surveillance of medicines is access to information regarding the safety of over-the-counter (OTC) medicines. OTC medicines are available from a wide range of sources including pharmacies, supermarkets, health food stores, and the internet [4], and as such information regarding their use and safety can be problematic to access. In Europe, it is estimated that around 10% of all medicine sales are for OTC medicines [5], while in the US, 40% of older individuals regularly use OTC medicines [6]. While many countries have pharmacovigilance programs which require manufacturers to report ADRs, these tend to focus on the surveillance of prescription medicines and do not always include OTC and non-prescription medicines. Despite this, there is increasing awareness of the need for pharmacovigilance of both prescription and non-prescription medicines, and in 2006, safety reporting for OTC medicines in the US was amended to require manufacturer reporting of ADRs for OTC medicines [7].

Previous research indicates that spontaneous reporting for OTC medicines does occur to some extent; however, the extent of ADR reporting for OTC medicines appears highly variable. For example, a retrospective analysis of pediatric ADR reports in the French national pharmacovigilance dataset in 2018 found that almost one quarter of ADR reports for children involved an OTC medicine [8]. In contrast, a study of the extent of ADR for non-analgesic OTC medicines using data from the Agency for Medicinal Products and Medical Devices of Croatia, found that ADR reports for non-analgesic OTC medicines comprised only 4% of all ADR reports [9], and a study examining ADRs associated with self-medication using data from the French regional Midi-Pyrenees pharmacovigilance dataset reported that self-medication ADRs accounted for only 1.3% of all ADR reports [5]. While there is evidence that ADRs involving OTC medicines are being reported via spontaneous reporting mechanisms, evidence that ADR reports for OTC medicines may be a suitable data source for generation and detection of pharmacovigilance safety signals is limited. A study in which Korean spontaneous ADR reports were used to explore the safety of herbal medicines in older patients with chronic disease, signals indicating potential safety concerns with nine herbal products were observed [10].

Non-steroidal anti-inflammatory medicines (NSAID) are commonly used for the management of pain and inflammation and are one of the most commonly used therapeutic medicine classes worldwide [11]. Reflecting their widespread use, NSAIDs are available in many countries as both prescription and OTC products. Gastrointestinal bleeding is a well-documented ADR associated with NSAID use, and a systematic review and meta-analysis of 18 cohort and case control studies reported that NSAID use was associated with a four-fold increase in the risk of upper gastrointestinal (GI) bleeding or perforation [12]. Most studies of NSAID-associated ADRS have focused on the use of prescription, rather than OTC, products; however, there is some concern that OTC NSAIDs may have a similar safety profile in terms of gastrointestinal ADRs. The Australian Regulatory body, the Therapeutics Goods Administration (TGA), conducted a review of the safety of NSAIDs in 2014, finding that the risks associated with prescription NSAIDs were also likely to apply to OTC NSAIDs. In the report, the TGA concluded that increased awareness of the risks associated with OTC NSAIDS among health professionals and consumers is needed [13]. The strength of the association between NSAID use and GI complications, and the widespread OTC availability of NSAIDs, makes this an appropriate scenario for exploring the potential of spontaneous ADR reporting data for safety signal detection of OTC medicines.

Traditionally, pharmacovigilance for OTC medicines has relied on prospective data collection, often using community pharmacies, which is time and resource intensive [14]. Spontaneous ADR reports represent a rich potential data source for the detection of safety signals associated with OTC medicines. Therefore, the aim of this study was to evaluate the potential for detecting safety signals for OTC medicines in National ADR spontaneous reporting data, using over-the-counter NSAIDs and gastrointestinal bleeding as an example.

## 2. Materials and Methods

### 2.1. Setting

This study used data from the Australian Adverse Drug Reactions System (ADRS) dataset and the Health Canada’s Canadian Vigilance Adverse Reaction Online Database (VAROD). Australia and Canada have comparable health systems and both countries have well-established pharmacovigilance systems, including well-developed spontaneous reporting mechanisms and databases. While the health systems and reporting systems are comparable between Australia and Canada, the datasets cannot be pooled due to heterogeneity in the structure and content, and thus the analyses were conducted separately in each dataset.

### 2.2. Data Sources

Given that the 2014 TGA review regarding the safety of NSAIDs may have increased awareness and reporting of ADRs associated with OTC NSAIDs in Australia, in this study, we focused on the detection of safety signals prior to the publication of the 2014 TGA report.

#### 2.2.1. Australian Adverse Drug Reactions System (ADRS) Dataset

All ADR reports from database inception (1971) to 2008 were included in the analysis. At the time of analysis, TGA data between 2008 and 2013 were not available.

All medicines in the ADRS dataset are coded using the World Health Organization’s Anatomical Therapeutic Chemical (ATC) classification [15], and all ADRs are classified using Medical Dictionary for Regulatory Activities (MedDRA) terminology to classify adverse reactions [16].

#### 2.2.2. Canadian Vigilance Adverse Reaction Online Database (VAROD)

All ADR reports from database inception (1965) to 2013 were included in the analysis. As in the Australian dataset, ADR reports in the Canadian dataset are coded using MedDRA terminology; however, it should be noted that medicines in the Canadian dataset are not coded according to the ATC classification system.

### 2.3. NSAID Exposure

#### 2.3.1. All NSAIDs

NSAIDs were defined as all medicines belonging to the ATC classes M01 available on the Australian and/or Canadian market during the time period covered by the data extracts. Topical NSAIDs (ATC class M02) were excluded from the analysis. No parenteral NSAIDS were on the market in either country during the relevant time period.

ATC codes were used to identify ADR reports involving NSAIDS in the Australian dataset, and free text fragments of brand and/or generic names were used to identify NSAID-associated reports in the Canadian dataset. Within each dataset, all NSAIDs were then classified as either a prescription or OTC product as described below.

#### 2.3.2. Prescription NSAIDs

Prescription NSAIDs were identified using commercial lists of all known product brand names and relevant medication schedules available in each country during the relevant time period.

#### 2.3.3. OTC NSAIDs

The same procedure was followed for OTC NSAIDs which were identified using commercial lists of all known over-the-counter product brand names and relevant medication schedules available in each country during the relevant time period. If no brand name was mentioned in the report, the medication was included in analyses for all NSAIDs but not included in the sub-group analyses of OTC and prescription NSAIDs. The systemic OTC NSAIDs and maximum product strength available OTC in each country during the study period are shown in Table 1.

#### 2.3.4. Gastrointestinal Bleeding Outcomes

Standardized MedDRA Queries (SMQs) associated with gastrointestinal perforation, ulceration, hemorrhage, or obstruction (SMQ 20000103) were used to identify relevant ADRs in each dataset [16]. The full list of terms and definitions included in the SMQs used in this analysis is included as Supplementary Materials. The presence of any preferred terms (PTs) within a report defined a case. Controls were reports which did not document the use of an NSAID.

### 2.4. Analyses

Disproportionality analyses were conducted as per Bate and Evans [19] and reporting odds ratios calculated as per Moore [20], using the exposure and outcome definitions above. In this method, the proportion of gastrointestinal bleeding reports in which a NSAID is reported is compared to the proportion of gastrointestinal bleeding reports for all other medicines, meaning gastrointestinal bleeding reports for all other medicines.

Analyses were conducted separately for all NSAIDS, prescription NSAIDs, and OTC NSAIDS, and analyses of the risk associated with individual NSAIDs limited to those agents available as OTC products during the study period as per Table 1. As age and sex were expected to be strongly associated with medication use, they were forced into the model at each step. Medications known to increase the risk of bleeding (ATC class N06AB: selective serotonin reuptake inhibitors, ATC class B01A: antithrombotic agents, and ATC class H02: systemic corticosteroids) as well as medications used for the management of conditions associated with an increased risk of bleeding (ATC class A02: drugs for acid-related disorders) were included as covariates in the model. Data manipulation and statistical analyses were conducted using MS Access^®^ (version 2013 Professional) and SPSS^®^ for Windows 7^®^ (version 20.0; SPSS Inc., Chicago, IL, USA).

### 2.5. Data Access and Ethical Approval

Australian Adverse Drug Reactions Systems data were provided by the Therapeutics Goods Administration, and the Canadian Vigilance Adverse Reaction database is publicly available online from the Canadian Government [21]. As this study used existing non-identifiable datasets and was considered to carry a negligible risk, it was exempted from ethical review under the Australian National Statement on Ethical Conduct in Human Research [22].

## 3. Results

The Australian dataset comprised a total of 202,833 ADR reports and the Canadian dataset 384,964 reports (Table 2). Approximately 13.0% of Australian reports (n = 26,369) and 12.0% (n = 46,196) of Canadian reports involved a NSAID. Of the reports involving NSAIDs, 15.3% (n = 4033/26,369) in the Australian dataset and 36.2% in the Canadian dataset involved OTC NSAIDs.

### 3.1. Safety of OTC NSAIDs

Safety signals indicating an increased risk of gastrointestinal bleeding with the use of NSAID were detected in both the Australian and Canadian datasets. Looking at unadjusted reporting odds ratios (Table 3), the use of any NSAID was associated with a nine-fold increase in the odds of experiencing gastrointestinal bleeding in the Australian dataset and a five-fold increase in the Canadian dataset (Table 3). After adjusting for age, gender, and medications known to increase the risk of bleeding and medications used for the management of conditions associated with an increased risk of bleeding, the reporting odds ratios reduced slightly to 7.6 in the Australian dataset and 3.5 in the Canadian dataset.

Safety signals for OTC NSAIDs were also consistently detected across both datasets indicating that the use of OTC NSAIDs is associated with an increased risk of gastrointestinal bleeding. The unadjusted RORs estimate a three- to four-fold risk across the Canadian and Australian datasets, respectively. After adjusting for age, gender, medications known to increase the risk of bleeding, and medications used for the management of conditions associated with an increased risk of bleeding, the signal weakened slightly. However, a two-fold increase in the risk of GI bleeding associated with OTC NSAID use remained across both datasets.

### 3.2. Safety Signals for Individual OTC NSAIDs

While OTC diclofenac was marketed in Australia during the study period, no reports involving OTC diclofenac were identified in the Australian dataset and, therefore, the reporting odds ratio for diclofenac was not calculated.

Looking at safety signals for the individual NSAIDs, all OTC NSAIDs included in the ADR reports were associated with an increased risk of GI bleeding. However, reports for the individual NSAIDs and the risk estimates for GI bleeding associated with them, varied between the Australian and Canadian datasets (Table 4). The risk estimates for GI bleeding for OTC NSAIDs were lower than the risk estimates for all (prescription and OTC) NSAIDs with the exception of ibuprofen in the Australian dataset (Table 4). OTC naproxen was associated with the highest risk of GI bleeding and OTC aspirin was associated with the lowest risk of GI bleeding in both the Australian and Canadian datasets.

## 4. Discussion

In this study, we show that spontaneous ADR reporting data can be used to detect safety signals and monitor the safety of OTC NSAIDs. We found that OTC NSAID use was associated with a two- to three-fold increase in the risk of a gastrointestinal bleeding and that the signal was consistent across both the Australian and Canadian National Pharmacovigilance datasets. Looking at the individual OTC NSAIDs, OTC aspirin, OTC ibuprofen, and OTC naproxen were all associated with an increased risk of GI bleeding, and among the individual NSAIDs, the highest risk of GI bleeding was associated with OTC naproxen and the lowest risk with OTC aspirin.

Our findings indicated that spontaneous ADR report data can be used to monitor the safety of OTC medicines. These findings build on a small but growing body of evidence supporting the use of voluntary reporting data for pharmacovigilance of OTC and non-prescription medicines. Similar signals for OTC medicines have been reported in a large study using the Japanese Adverse Event Report Database where safety signals for OTC analgesic and antipyretic agents [23], as well as OTC cough and cold medicines [24] were detected. Looking internationally, safety signals for OTC medicines have been reported in the Dutch Lareb dataset [2], and the findings from our study extend the evidence supporting the use of spontaneous ADR reporting data for pharmacovigilance of OTC medicines, to include the Asia Pacific and North American regions.

While the aim of this study was to explore the use of spontaneous ADR reporting data for monitoring the safety of OTC medicines, the risk estimates obtained in this study reflect those regarding the gastrointestinal safety of NSAIDs found in the wider pharmacovigilance literature. In our study, the highest risk of GI bleeding was associated with the use of OTC naproxen, and the lowest risk was observed with OTC aspirin and our risk estimates are similar to those reported in a systematic review and meta-analysis of 28 studies exploring the association between GI complications associated with NSAID use [25].

We observed a possible dose–response relationship between NSAID use and GI complications. For the majority of OTC NSAIDs, the risk of GI bleeding was higher with the use of all NSAIDs, which included both prescription and non-prescription agents, than with OTC NSAIDs. Internationally, OTC NSAIDs tend to be available in lower strengths and used in lower doses than prescription NSAIDs [26], and thus, as we observed, it is expected that a higher risk would be associated with prescription NSAIDs use due to the use of higher product strengths and dosages rather than non-prescription use.

One of the challenges with all adverse drug reaction reporting programs which rely on voluntary reporting, is the under-reporting of potential ADRs [27]. OTC medications are often used for self-medication, and as such, opportunities for a health professional follow-up of potential ADRs may be limited, which may further result in under-reporting. Involvement of patients and carers in ADR reporting is one solution that has been proposed to optimize reporting rates and ensure that ADRs involving OTC medicines are reported. However, the extent of patient reports in national datasets is likely to be low. A study of the extent of ADR reporting by patients in the Dutch Lareb dataset found only 61 reports for OTC medicines among the 1691 reports included in the study, and of these, only eight were made directly by patients [2]. This indicates that strategies to improve the reporting of OTC medicines targeting both patients and health professionals are needed.

### Strengths and Limitations

A strength of this study was the use of spontaneous reporting data from two distinct geographical locations. While there were differences in the specific risk estimates obtained between the two datasets, the risk estimates obtained were consistent with those reported in studies examining the safety of non-OTC NSAIDs, which indicates the robustness of the findings. The results also confirm what is known regarding the safety of NSAIDs; however, differences in the structure of the two datasets precluded a combination of the datasets. Future research to implement a common data model, for example, the one used by the WHO in the Vigibase ADR dataset, at the national level would allow future analysis of pooled data.

Like all studies, this study had a number of limitations. Spontaneous ADR reporting datasets rely on voluntary reporting of adverse reactions and cannot be used to determine the prevalence of particular outcomes. As discussed above, under-reporting is a well-known issue with adverse reaction reporting [27], and thus, we used disproportionality analyses for signal detection to take this into consideration. A further limitation is that we adjusted our analysis for a very limited set of potential confounders which were available within our datasets, and this is likely to impact the accuracy of the adjusted estimates obtained. Finally, as GI complications and bleeding are a well-documented ADR associated with the use of NSAIDs, there may be under-reporting by the health professionals and patients compared with other less known ADRs, which may reduce risk estimates based on proportionality.

## 5. Conclusions

This study demonstrates that safety signals for OTC medicines can be detected in spontaneous ADR reporting data. The detection of safety signals, especially for new medicines or previously unrecognized ADRs, is the first step in ensuring post-marketing safety of medicines. It should be noted that signal detection does not imply causality, and in-depth assessment, including confirmation from other data sources, should be undertaken once a signal is detected. Given the limitations, in terms of time and resources, of prospective data collection for pharmacovigilance, and the increasing availability of OTC medicines, there is a strong need for robust pharmacovigilance methods suitable for monitoring the post-market safety of OTC medicines, and that national ADR datasets could provide one option for pharmacovigilance and monitoring the safety of OTC medicines.

## Figures and Tables

**Table 1:** Systemic non-steroidal anti-inflammatory drugs (NSAIDs) and the maximum strength per dosage form available over-the-counter (OTC) medicines in Australia and Canada during the study period.

OTC NSAID	Maximum Product Strength Available OTC (Maximum Dose (mg) Per Dosage Form)	
	Australia (1971–2008) [17]	Canada (1965–2013) [18]
Aspirin	≤500 mg *	≤500 mg *
Diclofenac	≤25 mg	Not available OTC
Ibuprofen	≤200 mg	≤400 mg immediate release≤600 mg modified release
Naproxen	≤250 mg	≤200 mg

* All aspirin products are OTC.

**Table 2:** Characteristics of the Australian Adverse Drug Reactions Systems and Canadian Vigilance Adverse Reaction data included in the analysis.

	Australia n (%)	Canada n (%)
Time period covered	1971–2008	1965–2013
Adverse drug reaction (ADR) reports	202,833 (100%)	384,964 (100%)
Reports with any NSAID	26,369 (13.0%)	46,196 (12.0%)
Reports with prescription NSAIDs	11,298 (5.6%)	12,065 (3.1%)
Reports with OTC NSAIDs	4033 (2.0%)	16,723 (4.3%)
Reports of gastrointestinal bleeding	2421 (1.2%)	6001 (1.6%)

**Table 3:** Risk of gastrointestinal (GI) bleeding or hemorrhage associated with the use of all NSAIDs, prescription NSAIDs, and OTC NSAIDs in Australia and Canada.

Australia (n = 202,833)							
*NSAID Status*	Number of Reports for GI Bleeding	Unadjusted			Adjusted *		
		Reporting Odds Ratio	95% Confidence Interval		Reporting Odds Ratio	95% Confidence Interval	
			Lower	Upper		Lower	Upper
All ^§^	1410	9.449	8.706	10.254	7.619	7.003	8.289
Prescription	751	8.095	7.411	8.843	7.366	6.725	8.068
OTC	183	4.175	3.579	4.870	2.847	2.432	3.332
**Canada (n = 384,964)**							
	**Number of Reports for GI Bleeding**	**Unadjusted**			Adjusted *		
		**Reporting Odds Ratio**	**95% Confidence Interval**		**Reporting Odds Ratio**	**95% Confidence Interval**	
			**Lower**	**Upper**		**Lower**	**Upper**
All ^§^	2289	4.598	4.361	4.849	3.712	3.508	3.928
Prescription	646	3.883	3.571	4.222	3.010	2.755	3.288
OTC	764	3.318	3.071	3.586	2.322	2.141	2.519

* Adjusted for age, gender, medications known to increase the risk of bleeding (Anatomical Therapeutic Chemical (ATC) class N06AB: selective serotonin reuptake inhibitors, ATC class B01A: antithrombotic agents, and ATC class H02: systemic corticosteroids) as well as medications used for the management of conditions associated with an increased risk of bleeding (ATC class A02: drugs for acid-related disorders). ^§^ All NSAIDs includes all Prescription NSAIDs, all OTC NSAIDs, and all NSAIDs that could not be classified accurately as either Prescription or OTC.

**Table 4:** Risk of GI bleeding/hemorrhage reported with the use of individual OTC NSAIDs in Australia and Canada.

Australia (n = 202,833)								
OTC NSAID	Regulatory Status	Number of Reports for GI Bleeding	Unadjusted			Adjusted *		
			Reporting Odds Ratio	95% Confidence Interval		Reporting Odds Ratio	95% Confidence Interval	
				Lower	Upper		Lower	Upper
Aspirin	All^§^	514	6.244	5.651	6.900	3.739	3.362	4.159
	OTC	156	4.152	3.518	4.901	2.589	2.185	3.068
Ibuprofen	All^§^	85	4.796	3.840	5.990	5.210	4.156	6.532
	OTC	23	4.679	3.068	7.135	6.370	4.127	9.832
Naproxen	All^§^	200	8.246	7.092	9.588	8.524	7.306	9.945
	OTC	2	2.269	0.557	9.250	4.363	1.067	17.846
**Canada (n = 384,964)**								
**OTC NSAID**	**Regulatory Status**	**Number of Reports for GI Bleeding**	**Unadjusted**			Adjusted *		
			**Reporting Odds Ratio**	**95% Confidence Interval**		**Reporting Odds Ratio**	**95% Confidence Interval**	
				**Lower**	**Upper**		**Lower**	**Upper**
Aspirin	All ^§^	1227	3.744	3.512	3.992	2.614	2.436	2.804
	OTC	479	2.949	2.681	3.244	1.761	1.590	1.951
Ibuprofen	All ^§^	176	2.557	2.194	2.979	2.696	2.295	3.167
	OTC	90	2.556	2.067	3.161	2.628	2.101	3.287
Naproxen	All ^§^	284	4.564	4.036	5.162	3.793	3.328	4.322
	OTC	272	4.628	4.082	5.248	3.798	3.325	4.339

* Adjusted for age, gender, medications known to increase the risk of bleeding (ATC class N06AB: selective serotonin reuptake inhibitors, ATC class B01A: antithrombotic agents, and ATC class H02: systemic corticosteroids) as well as medications used for the management of conditions associated with an increased risk of bleeding (ATC class A02: drugs for acid-related disorders). ^§^ All includes all Prescription, OTC, and NSAIDs which were unable to be accurately classified as Prescription or OTC.

## Supplementary Materials

The following are available online at https://www.mdpi.com/2226-4787/8/3/174/s1, Table S1: Standardised MedDRA Query terms (SMQs), preferred terms and MEDRA codes for Gastrointestinal perforation, ulceration, hemorrhage or obstruction (SMQ 20000103).
