# Supplementary material for: Potential for Detection of Safety Signals for Over-the-Counter Medicines Using National ADR Spontaneous Reporting Data: The Example of OTC NSAID-Associated Gastrointestinal Bleeding
Source: Pharmacy (Basel). 2020 Sep 17;8(3):174. doi: 10.3390/pharmacy8030174 (PMC7558367; doi:10.3390/pharmacy8030174)
Supplement: Supplementary file 1 [file pharmacy-08-00174-s001.pdf]

Article

Potential for Detection of Safety Signals for Over the Counter Medicines Using National ADR Spontaneous Reporting Data: The Example of OTC NSAID Associated Gastrointestinal Bleed

Elina Amatya [1], Romano Fois [2], Kylie A. Williams [3] and Lisa G. Pont [3,*]

[1] eHealth NSW, Chatswood NSW 2067, Australia; sojelina@gmail.com
[2] Ferring Pharmaceuticals, Pymble, NSW 2073, Australia; romanof015@gmail.com
[3] Discipline of Pharmacy, Graduate School of Health, University of Technology Sydney, Sydney NSW 2007, Australia; kylie.williams@uts.edu.au
* Correspondence: lisa.pont@uts.edu.au

Supplementary Materials

Table 1. Standardised MedDRA Query terms (SMQs), preferred terms and MEDRA codes for Gastrointestinal perforation, ulceration, hemorrhage or obstruction (SMQ 20000103).

SMQ	Preferred term	MedDRA Code
SMQ Gastrointestinal perforation, ulcer, haemorrhage, obstruction non-specific findings/procedures (SMQ) [20000104]	Abdominal discomfort	10000059
	Bloody peritoneal effluent	10067442
	Colonoscopy abnormal	10010008
	Endoscopy large bowel abnormal	10014810
	Endoscopy small intestine abnormal	10014817
	Endoscopy upper gastrointestinal tract abnormal	10014820
	Enterocolitis	10014893
	Enterostomy	10057005
	Enterostomy closure	10057074
	Epigastric discomfort	10053155
	Explorative laparotomy	10053361
	Gastrectomy	10061965
	Gastric atony	10017753
	Gastric bypass	10061966
	Gastric hypomotility	10052405
	Gastric ileus	10058035
	Gastric operation	10061969
	Gastric volvulus	10052541
	Gastro-enterostomy	10017873
	Gastrointestinal pain	10017999
	Gastrointestinal sounds abnormal	10067715
	Haemorrhagic ascites	10059766
	Ileal operation	10062010
	Ileectomy	10057006
	Ileocolic bypass	10068066
	Ileostomy	10021321
	Ileus	10021328
	Ileus paralytic	10021333
	Ileus spastic	10021335
	Impaired gastric emptying	10021518

	Intestinal resection	10054193
	Intra-abdominal haemorrhage	10061249
	Intussusception	10022863
	Jejunal operation	10062027
	Jejunectomy	10057007
	Jejunostomy	10023180
	Laparoscopy abnormal	10023694
	Mechanical ileus	10051399
	Meconium peritonitis	10058113
	Mouth cyst	10028020
	Mucosal erosion	10061297
	Mucosal haemorrhage	10061298
	Mucosal ulceration	10028124
	Oesophageal discomfort	10053634
	Oesophageal hypomotility	10067752
	Oesophageal pain	10030180
	Oesophagoenterostomy	10030221
	Oesophagoscopy abnormal	10030223
	Peritoneal haematoma	10058095
	Peritoneal haemorrhage	10034666
	Proctocolitis	10036784
	Proctoscopy abnormal	10036787
	Proctosigmoidoscopy abnormal	10053286
	Rectal ultrasound abnormal	10056621
	Retroperitoneal haematoma	10058360
	Retroperitoneal haemorrhage	10038980
	Sigmoidoscopy abnormal	10040670
	Subileus	10050396
	Toxic dilatation of intestine	10044145
	Volvulus	10047697
	Vomiting projectile	10047708
	X-ray gastrointestinal tract abnormal	10061582
	X-ray with contrast lower gastrointestinal tract abnormal	10059705
	**X-ray with contrast upper gastrointestinal tract abnormal**	10059707
	PT	MedDRA Code
SMQ Gastrointestinal perforation, ulcer, haemorrhage, obstruction non-specific findings/procedures (SMQ) [20000105]	Anal stenosis	10002176
	Anastomotic stenosis	10051268
	Anastomotic ulcer, obstructive	10002250
	Anorectal stenosis	10002581
	Barium impaction	10069083
	Colonic obstruction	10010000
	Colonic stenosis	10010004
	Distal ileal obstruction syndrome	10056361
	Distal intestinal obstruction syndrome	10056361
	Duodenal obstruction	10013830
	Duodenal scarring	10050188
	Duodenal stenosis	10050094
	Duodenal ulcer perforation, obstructive	10013850
	Duodenal ulcer, obstructive	10013855
	Fibrosing colonopathy	10052072
	Gastric stenosis	10061970

	Gastric ulcer haemorrhage, obstructive	10017829
	Gastric ulcer perforation, obstructive	10017836
	Gastric ulcer, obstructive	10017840
	Gastrointestinal anastomotic leak	10065879
	Gastrointestinal hypomotility	10052105
	Gastrointestinal motility disorder	10061173
	Gastrointestinal obstruction	10061974
	Gastrointestinal stenosis	10018007
	Ileal stenosis	10021307
	Impaired gastric emptying	10021518
	Intestinal obstruction	10022687
	Intestinal stenosis	10022699
	Jejunal stenosis	10023176
	Large intestinal obstruction	10062062
	Large intestinal obstruction reduction	10023794
	Large intestinal stricture	10023797
	Necrotising colitis	10051606
	Necrotising gastritis	10049150
	Necrotising oesophagitis	10055668
	Neonatal intestinal obstruction	10028951
	Obstruction gastric	10029957
	Oesophageal obstruction	10030178
	Oesophageal stenosis	10030194
	Peptic ulcer perforation, obstructive	10034358
	Peptic ulcer, obstructive	10034365
	Prepyloric stenosis	10050173
	Rectal obstruction	10065707
	Rectal stenosis	10038079
	Small intestinal obstruction	10041101
	Small intestinal stenosis	10062263
SMQ Gastrointestinal perforation, ulcer, haemorrhage, obstruction non-specific findings/procedures (SMQ) [20000106]	Anal erosion	10067272
	Anal ulcer	10002180
	Anal ulcer haemorrhage	10063896
	Anastomotic ulcer	10002243
	Anastomotic ulcer haemorrhage	10002244
	Anastomotic ulcer perforation	10002248
	Anorectal ulcer	10002582
	Colitis erosive	10058358
	Colitis ulcerative	10009900
	Duodenal scarring	10050188
	Duodenal ulcer	10013836
	Duodenal ulcer haemorrhage	10013839
	Duodenal ulcer perforation	10013849
	Duodenal ulcer, obstructive	10013855
	Erosive duodenitis	10062532
	Erosive oesophagitis	10063655
	Gastric ulcer	10017822
	Gastric ulcer haemorrhage	10017826
	Gastric ulcer haemorrhage, obstructive	10017829
	Gastric ulcer helicobacter	10051348
	Gastric ulcer perforation	10017835
	Gastric ulcer perforation, obstructive	10017836
	Gastric ulcer surgery	10057348

	Gastritis erosive	10057348
	Gastritis haemorrhagic	10017866
	Gastritis hypertrophic	10017868
	Gastroduodenal ulcer	10017886
	Gastro-enterostomy	10017873
	Gastrointestinal anastomotic leak	10065879
	Gastrointestinal erosion	10060709
	Gastrointestinal pain	10017999
	Gastrointestinal ulcer	10061459
	Gastrointestinal ulcer haemorrhage	10056743
	Gastrointestinal ulcer management	10057161
	Gastrointestinal ulcer perforation	10061975
	Gingival erosion	10018282
	Haemorrhagic erosive gastritis	10067786
	Ileal ulcer	10021309
	Ileal ulcer perforation	10021310
	Intestinal ulcer	10022714
	Intestinal ulcer perforation	10061248
	Ischaemic ulcer	10059084
	Jejunal ulcer	10023177
	Jejunal ulcer perforation	10023178
	Large intestinal ulcer	10023799
	Large intestinal ulcer haemorrhage	10061262
	Lip erosion	10051992
	Mouth ulceration	10028034
	Necrotising colitis	10051606
	Necrotising gastritis	10049150
	Necrotising oesophagitis	10055668
	Oesophageal ulcer	10030201
	Oesophageal ulcer haemorrhage	10030202
	Oesophageal ulcer perforation	10052488
	Oesophagitis ulcerative	10049098
	Oesophagoscopy abnormal	10030223
	Peptic ulcer	10034341
	Peptic ulcer haemorrhage	10034344
	Peptic ulcer perforation	10034354
	Peptic ulcer perforation, obstructive	10034358
	Peptic ulcer reactivated	10034359
	Peptic ulcer, obstructive	10034365
	Perforated duodenal ulcer repair	10034390
	Perforated peptic ulcer oversewing	10034397
	Perforated ulcer	10062065
	Pharyngeal erosion	10062773
	Proctitis ulcerative	10036783
	Prophylaxis against gastrointestinal ulcer	10054981
	Rectal ulcer	10038080
	Rectal ulcer haemorrhage	10038081
	Sigmoidoscopy abnormal	10040670
	Small intestinal ulcer haemorrhage	10061550
	Small intestine ulcer	10041133
	Stomatitis	10042128
	Stomatitis haemorrhagic	10042132
	Stress ulcer	10042220
	Ulcer	10045285
	Ulcer haemorrhage	10061577

SMQ Gastrointestinal perforation, ulcer, haemorrhage, obstruction non-specific findings/procedures (SMQ) [20000107]	Abdominal abscess	10060921
	Abdominal wall abscess	10000099
	Abscess intestinal	10000285
	Acquired tracheo-oesophageal fistula	10000582
	Anal abscess	10048946
	Anal fistula	10002156
	Anal fistula excision	10002157
	Anastomotic ulcer perforation	10002248
	Anovulvar fistula	10050362
	Aorto-duodenal fistula	10002924
	Aorto-oesophageal fistula	10066870
	Appendiceal abscess	10049764
	Appendicitis perforated	10003012
	Colon fistula repair	10052931
	Colonic fistula	10009995
	Diverticular fistula	10013536
	Diverticular perforation	10061820
	Douglas' abscess	10049583
	Duodenal fistula	10013828
	Duodenal perforation	10013832
	Duodenal ulcer perforation	10013849
	Duodenal ulcer perforation, obstructive	10013850
	Enterocolonic fistula	10056991
	Enterocutaneous fistula	10051425
	Enterovesical fistula	10062570
	Gastric fistula	10065713
	Gastric perforation	10017815
	Gastric ulcer perforation	10017835
	Gastric ulcer perforation, obstructive	10017836
	Gastrointestinal anastomotic leak	10065879
	Gastrointestinal fistula	10017877
	Gastrointestinal perforation	10018001
	Gastrointestinal ulcer perforation	10061975
	Gastropleural fistula	10067091
	Ileal perforation	10021305
	Ileal ulcer perforation	10021310
	Ileorectal fistula	10056993
	Intestinal fistula	10022647
	Intestinal fistula repair	10052991
	Intestinal perforation	10022694
	Intestinal ulcer perforation	10061248
	Jejunal perforation	10023174
	Jejunal ulcer perforation	10023178
	Large intestine perforation	10023804
	Oesophageal fistula repair	10058381
	Oesophageal perforation	10030181
	Oesophageal rupture	10052211
	Oesophageal ulcer perforation	10052488
	Oesophagobronchial fistula	10056992
	Paraoesophageal abscess	10056086
	Peptic ulcer perforation	10034354
	Peptic ulcer perforation, obstructive	10034358
	Perforated duodenal ulcer repair	10034390
	Perforated peptic ulcer oversewing	10034397

	Perforated ulcer	10062065
	Perineal abscess	10052457
	Peritonitis	10034674
	Peritonitis bacterial	10062070
	Rectal abscess	10048947
	Rectal fistula repair	10053267
	Rectal perforation	10038073
	Rectourethral fistula	10066892
	Retroperitoneal abscess	10038975
	Small intestinal perforation	10041103
SMQ Gastrointestinal haemorrhage (SMQ) [20000108]	Anal haemorrhage	10049555
	Anal ulcer haemorrhage	10063896
	Anastomotic haemorrhage	10056346
	Anastomotic ulcer haemorrhage	10002244
	Chronic gastrointestinal bleeding	10050399
	Colonic haematoma	10009996
	Diarrhoea haemorrhagic	10012741
	Diverticulitis intestinal haemorrhagic	10013541
	Duodenal operation	10061826
	Duodenal ulcer haemorrhage	10013839
	Duodenitis haemorrhagic	10012741
	Enterocolitis haemorrhagic	10014896
	Gastric haemorrhage	10017788
	Gastric occult blood positive	10067855
	Gastric operation	10061969
	Gastric ulcer haemorrhage	10017826
	Gastric ulcer haemorrhage, obstructive	10017829
	Gastric varices haemorrhage	10057572
	Gastritis alcoholic haemorrhagic	10017857
	Gastritis haemorrhagic	10017866
	Gastroduodenal haemorrhage	10053768
	Gastroduodenitis haemorrhagic	10048712
	Gastrointestinal anastomotic leak	10065879
	Gastrointestinal haemorrhage	10017955
	Gastrointestinal ulcer haemorrhage	10056743
	Haematemesis	10018830
	Haematochezia	10018836
	Haemorrhagic erosive gastritis	10067786
	Haemorrhoidal haemorrhage	10054787
	Intestinal haemorrhage	10059175
	Intra-abdominal haematoma	10056457
	Large intestinal haemorrhage	10052534
	Large intestinal ulcer haemorrhage	10061262
	Lower gastrointestinal haemorrhage	10050953
	Mallory-Weiss syndrome	10026712
	Melaena	10027141
	Melaena neonatal	10049777
	Mouth haemorrhage	10028024
	Occult blood positive	10061880
	Oesophageal haemorrhage	10030172
	Oesophageal ulcer haemorrhage	10030202
	Oesophageal varices haemorrhage	10030210
	Oesophagitis haemorrhagic	10030219
	Oesophagoscopy abnormal	10030223

	Peptic ulcer haemorrhage	10034344
	Proctitis haemorrhagic	10036778
	Rectal haemorrhage	10038063
	Rectal ulcer haemorrhage	10038081
	Small intestinal haemorrhage	10052535
	Small intestinal ulcer haemorrhage	10061550
	Stomatitis haemorrhagic	10042132
	Ulcer haemorrhage	10061577
	Upper gastrointestinal haemorrhage	10046274
